# Systematic analysis of the gerontome reveals links between aging and age-related diseases

**DOI:** 10.1093/hmg/ddw307

**Published:** 2016-09-04

**Authors:** Maria Fernandes, Cen Wan, Robi Tacutu, Diogo Barardo, Ashish Rajput, Jingwei Wang, Harikrishnan Thoppil, Daniel Thornton, Chenhao Yang, Alex Freitas, João Pedro de Magalhães

**Affiliations:** 1 Integrative Genomics of Ageing Group, Institute of Ageing and Chronic Disease, University of Liverpool, Liverpool, UK; 2 LaSIGE - Large-Scale Informatics Systems Laboratory, Faculty of Sciences, University of Lisbon, Portugal; 3 School of Computing, University of Kent, Canterbury, UK; 4 Department of Computer Science, University College London, London, UK; 5 Research Group for Computational Systems Biology, German Center for Neurodegenerative Diseases (DZNE), Göttingen, Germany

## Abstract

In model organisms, over 2,000 genes have been shown to modulate aging, the collection of which we call the ‘gerontome’. Although some individual aging-related genes have been the subject of intense scrutiny, their analysis as a whole has been limited. In particular, the genetic interaction of aging and age-related pathologies remain a subject of debate. In this work, we perform a systematic analysis of the gerontome across species, including human aging-related genes. First, by classifying aging-related genes as pro- or anti-longevity, we define distinct pathways and genes that modulate aging in different ways. Our subsequent comparison of aging-related genes with age-related disease genes reveals species-specific effects with strong overlaps between aging and age-related diseases in mice, yet surprisingly few overlaps in lower model organisms. We discover that genetic links between aging and age-related diseases are due to a small fraction of aging-related genes which also tend to have a high network connectivity. Other insights from our systematic analysis include assessing how using datasets with genes more or less studied than average may result in biases, showing that age-related disease genes have faster molecular evolution rates and predicting new aging-related drugs based on drug-gene interaction data. Overall, this is the largest systems-level analysis of the genetics of aging to date and the first to discriminate anti- and pro-longevity genes, revealing new insights on aging-related genes as a whole and their interactions with age-related diseases.

## Introduction

Aging is a major social and medical challenge of the 21^st^ century. The most accepted mechanisms of aging include inflammation (1), apoptosis, oxidative stress, accumulation of DNA damage, cell cycle deregulation and mitochondrial dysfunction (2–4). In addition, one of the major breakthroughs in the field of aging research is the discovery that, in model organisms, aging is under genetic regulation (5). In the past 20 years, aging has been shown to be under genetic control in various short-lived model organisms, and in particular in yeast, worms, flies and mice. According to the GenAge database (6), over 2,000 genes can modulate aging and/or longevity in model organisms. We call the collection of these aging-related genes the ‘gerontome’ (7). Many of these genes work in common pathways (4), which include the insulin-like growth factor (IGF-1) signalling pathway, the target of rapamycin (TOR) pathway and the AMP kinase pathway (5).

Although some individual aging-related genes have been the subject of intense scrutiny, their analysis as a whole has been limited (8–11). Yet genes and proteins do not act individually. Therefore, biological networks provide a more realistic description of biological systems than single-molecule studies and give way to the integration of several types of data (12). Indeed, network analyses have already revealed insights on aging and its manipulation (13–15).

Aging is associated with various diseases. The main categories of aging-related pathologies are: cancer, cardiovascular diseases, neurodegenerative diseases, nutritional and metabolic diseases (16–18). The relationship between aging and age-related diseases has long been a contentious topic. A previous study has shown that the analysis of networks can uncover links between aging-related genes and age-related diseases (19), but many questions remain unanswered, like which aging-related genes and pathways are important in these interactions? Moreover, we have further classified aging-related genes as anti- or pro-longevity, depending on how they are genetically manipulated and whether they increase or decrease lifespan in model organisms (6). Whether and how anti- and pro-longevity genes interact with aging disease-related genes is unknown.

In this work, we performed a systematic analysis of the gerontome, the largest such analysis to date and the first to discriminate anti- and pro-longevity genes. Our analysis of pathways common to aging-related genes allows us to systematically classify pathways as anti- or pro-longevity, even though these mostly recapitulate previous findings. By contrast, our comparison of aging-related genes with age-related disease genes reveals several unexpected results: we found an association between aging-related genes and age-related diseases, yet this association is surprisingly organism-specific and driven by a small cluster of genes. Besides, one major issue in network analysis is whether some genes being better studied than others (what we call publication bias) impacts the underlying datasets and subsequent results. We correct for publication bias and show that a small but detectable fraction of results from protein-protein interaction network analysis is indeed influenced by whether genes are more studied than others. Lastly, we identify and rank drugs being targeted by aging-related genes which are promising for additional studies.

## Results

Our systematic analysis of the gerontome employed the GenAge database developed by our lab (6). This includes 298 human candidate aging-related genes and genes associated with aging and/or longevity in model organisms of which over 1,000 can be converted to human homologs (see Materials and Methods). Model organism aging-related genes were further classified as pro- or anti-longevity depending on their effects: pro-longevity genes are defined as genes whose decreased expression reduces lifespan and/or whose overexpression extends lifespan; accordingly, anti-longevity genes are those whose decreased expression extends lifespan and/or whose overexpression decreases it (6) (Materials and Methods). This work is the first to consider such classification in a systematic way.

### Processes and pathways overrepresented in pro- and anti-longevity genes

First, we performed a functional enrichment analysis of pro- and anti-longevity genes in each of the major model organisms. For pro-longevity genes, the most significant enriched pathways were p53-signalling pathway and cell cycle in mice; hypoxia response via HIF activation in *Drosophila melanogaster*; regulation of autophagy and oxidative phosphorylation in *C. elegans* (Supplementary Dataset 1). On the other hand, for anti-longevity genes, insulin signalling, growth hormone signalling and IGF-1 receptor pathways were overrepresented in mice; the PI3 kinase pathway, oxidative phosphorylation and IGF pathway in *Drosophila*; oxidative phosphorylation, mTOR signalling pathway in *C. elegans*; ribosome in *Saccharomyces cerevisiae*. Some pathways like mTOR signalling, autophagy, insulin signalling and ribosome were enriched in more than one model organism (Supplementary Dataset 1).

In addition to the more traditional functional enrichment, we also used a recently proposed feature selection method, from the area of data mining (or machine learning) to select relevant biological process Gene Ontology (GO) terms for predicting the pro-longevity or anti-longevity effect of a gene on a model organism (8). Among the top ranking GO terms identified by that feature selection method, terms associated with pro-longevity included apoptotic signalling pathway and cell cycle checkpoint in mice, lipid metabolic process in *Drosophila*, autophagy in *C. elegans* and telomere organization in *S. cerevisiae*. By contrast, top ranking GO terms associated with anti-longevity included positive regulation of multicellular organism growth in mice, sensory perception in *Drosophila* and translation in *C. elegans* (Supplementary Material, Dataset 2).

Although the two aforementioned methods work in very different ways, there is some overlap between their results. In particular, in the results for mice, both methods found terms related to insulin signalling or growth to be significantly associated with anti-longevity; and terms related to the cell cycle were found to be significantly associated with pro-longevity. In addition, some terms related to autophagy were found to be significantly associated with pro-longevity in *C. elegans* by both methods.

These results mostly recapitulate current knowledge of pathways associated with longevity manipulation in model organisms. Nonetheless, our results highlight pathways with pro- and anti-longevity effects and allow us to classify such pathways in a more consistent, systematic way.

### Pro- and anti-longevity networks are interwined

Next, we tested if aging-related genes interact with each other and if we can observe the differences between the ways that pro- and anti-longevity genes form protein interaction networks. To perform this analysis, we employed protein-protein interaction data from BioGRID (see Materials and Methods) and focused our attention on worm genes, as the dataset of aging-related genes in worms is by far the largest among the animal models in GenAge (Table 1).

**Table 1.** Number of genes plus average and median number of publications per gene in each dataset

**Dataset**^a^	Num. of genes	Average num. pubs.	Median num. pubs.
Human genome (NCBI)	20183^b^	8.7	6
Human interactome (BioGRID)	15000^c^	10.4	8
Human aging-related genes	298	30.3	23
All aging-related orthologs	894	14.5	10
anti-longevity	448	13.2	9
pro-longevity	421	15.9	11
*M. musculus*	84	26.8	19
anti-longevity	23	22.7	13
pro-longevity	59	28.6	21
*D. melanogaster*	135	19.9	13
anti-longevity	48	20.1	12
pro-longevity	87	19.6	13
*C. elegans*	693	13.1	9
anti-longevity	381	13.0	9
pro-longevity	290	13.1	9
*S. cerevisiae*	62	17.9	14
anti-longevity	41	14.7	10
pro-longevity	13	23.2	15

Notes:.

a All datasets refer to human genes, including human orthologs of genes from various model organisms.

b Genome has 20183 annotated genes in NCBI but only 19071 are in the Swiss-Prot database.

c Interactome has 15000 annotated genes in NCBI but only 14498 are in the Swiss-Prot database.

Out of all the worm genes classified as anti- or pro-longevity genes (*n =* 719), 283 genes had interactions in the BioGRID interactome (109 pro- and 174 anti-longevity genes). The average connectivity degree of pro-longevity genes was slightly higher than that of anti-longevity genes (8.42 compared to 5.43), and on average, both sets of aging-associated genes included more connected genes than similarly-sized random sets from the interactome (the connectivity degree for the entire interactome is 3.8). The clustering coefficient of pro-longevity genes was also higher than that of anti-longevity genes (0.108 compared to 0.063), showing that on average pro-longevity genes tend to cluster better than anti-longevity genes.

**Figure 1.**
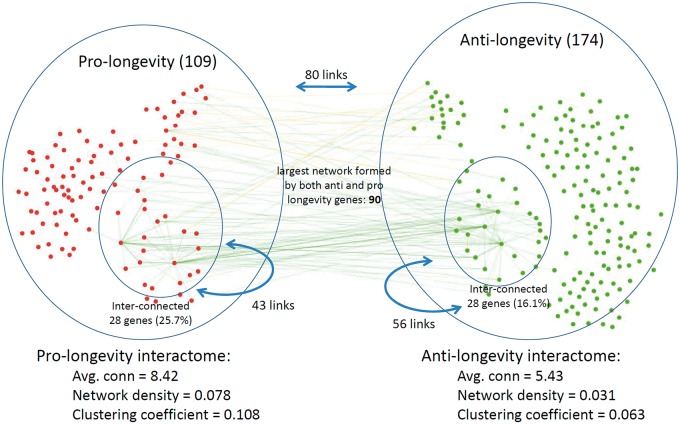
Protein–protein interactions between worm aging-related genes. Pro-longevity genes are depicted in red and anti-longevity genes in green. For each of the two gene sets, the smaller inside ellipse indicates genes that form a continuously connected network. Left right straight and curved arrows are used to summarize undirected interactions between genes from different and the same gene set, respectively.

In addition, we found that pro- and anti-longevity genes are much intertwined, with almost as many protein-protein interactions between genes from opposite categories (80 interactions) as between genes from the same category (43 interactions between pro-longevity genes and 56 interactions between anti-longevity genes) (Fig. 1). While pro- and anti-longevity genes can form two network cores by themselves (28 genes are interconnected for each set), they also form a much larger network when taken together (90 genes), suggesting that the way in which pro- and anti-longevity genes determine lifespan is in many cases dependent on one another.

Various previous studies have shown aging-related genes to form strong networks (9,10,14), as is normal in biology, but our results expand these observations to pro- and anti-longevity subnetworks and support substantial interactions between pro- and anti-longevity genes.

### Comparison with longevity-associated human genes

In addition to genetic manipulations in model organisms, a number of genes have been associated with longevity in human populations (20). We therefore also performed a functional enrichment analysis of these genes using data from the LongevityMap, which consists of 755 human genes, 328 of which associated with longevity in at least one genetic association study (20). For a first functional enrichment analysis using DAVID (see Materials and Methods), with the background set as default, 49 clusters showed an enrichment score greater than 2.5 (Supplementary Dataset 3). With the background set as LongevityMap genes, 62 clusters with an enrichment score > 2.5 were obtained (Supplementary Material, Dataset 3). A similar functional annotation clustering pattern as seen in the first run was observed (Supplementary Material, Fig. S1), and major enriched clusters consisted of: regulation of apoptosis, regulation of phosphorylation, response to environment, regulation of locomotion and response to hormone stimulus.

Results from human longevity-associated genes only modestly overlap with the above results for model organisms, although some pathways thought to be related to aging (e.g., apoptosis, response to oxidative stress and mTOR signalling) were found. Enriched clusters also included terms related to age-related diseases like cancer and diabetes mellitus (Supplementary Dataset 3). This may reflect how researchers choose candidate genes for longevity association studies, however. Perhaps researchers tend to select candidate genes for their studies that are suspected of playing important roles in human longevity, or in severe pathological processes that can significantly impair longevity.

### Overlap between aging-related genes and age-related diseases

Next we aimed to study the genetic overlap between aging and age-related diseases (ARDs). For this analysis, we used human genes associated with ARDs from public databases (see Materials and Methods), human candidate aging-related genes and human homologs of genes associated with aging in model organisms from GenAge (6). Common or shared genes between ARDs and aging gene sets are referred to as common aging and disease (CAD) genes. In addition to an analysis focused on individual age-related diseases, a set named ‘all diseases’ and another named ‘all classes’ were created, composed of all genes considered in the analyses per individual age-related disease and per age-related disease class, respectively (Materials and Methods).

**Figure 2.**
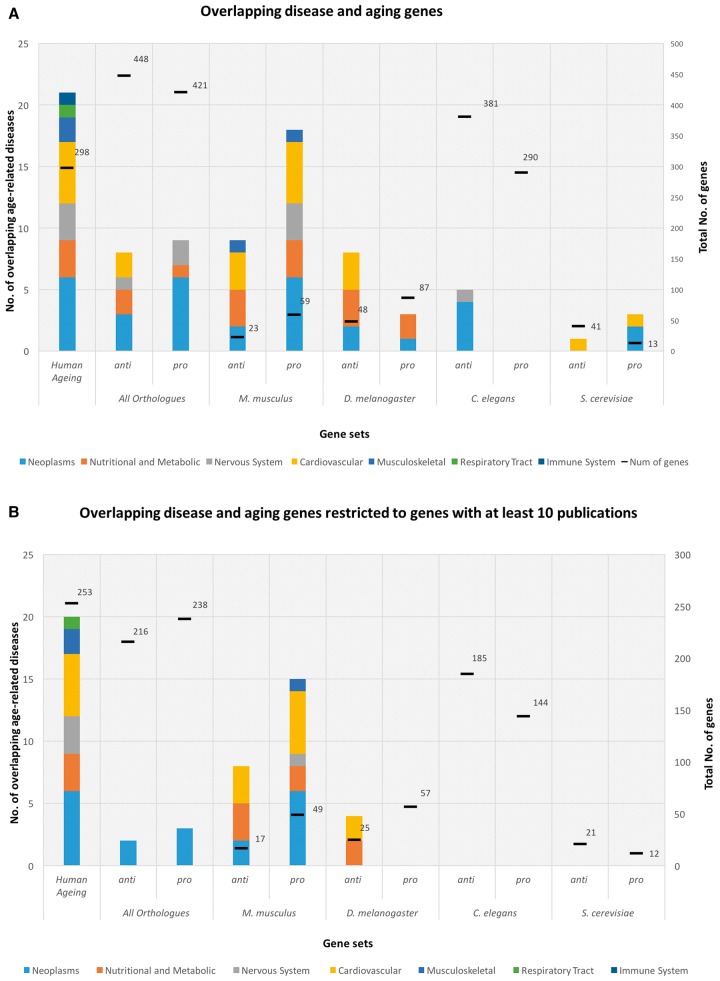
The y-axis quantifies the number of age-related diseases which significantly overlap with aging-related genes; the x-axis describes the aging-related gene sets studied according to the source organism (i.e., human plus human homologs of aging-related genes from each model organism). The columns have seven different colours to represent each age-related disease classe analysed: Neoplasms (light blue), Nutritional and Metabolic diseases (orange), Nervous System diseases (light grey), Cardiovascular diseases (yellow), Musculoskeletal diseases (blue), Respiratory Tract diseases (green) and Immune System diseases (dark blue). The first column represents the number of age-related diseases with a significant overlap with candidate human aging-associated genes. Model organisms are ordered by evolutionary proximity to humans. The genome was considered as background. The secondary y-axis displays the number of genes from the respective gene sets. (**A**) shows the number of significant overlapping aging-related genes with age-related diseases. (**B**) shows the number of significant overlapping aging-related genes with age-related diseases with PBC (i.e., only genes with more than 10 publications were used).

**Figure 3.**
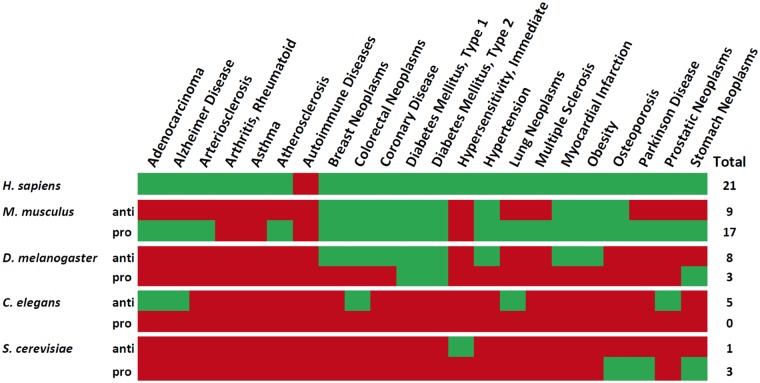
Overlapping aging-related genes for various organisms with age-related disease genes sets. Green means significant overlap between aging-related and age-related disease genes and red means there is no significant overlap. Model organisms are in descending order of their proximity to humans.

As expected, the human aging-related gene set has the most associations with ARD genes. In addition, the immune system and respiratory tract disease classes only show a relation with aging in human aging-related genes (Fig. 2A). Among the human homologs of genes associated with aging in model organisms, the musculoskeletal disease class only exhibits a significant overlap with aging-related genes in the mouse. A decrease in the overlap between aging and age-related diseases as evolutionary distance increases is also clear from our results (Fig. 2A) with the mouse showing an overlap with more ARDs when compared with the other model organisms, even though fewer aging-related genes are known in mice than in flies or worms. This is also clear from looking at individual ARDs (Fig. 3) since in mice there is a significant overlap with 9 and 17 ARDs for anti- and pro-longevity sets, respectively, while the remaining model organisms present the following values: *Drosophila* - 8 and 3; *C. elegans* - 5 and 0; and finally *S. Berevisiae* - 1 and 3.

We also compared overlaps between anti- and pro-longevity genes. Pro-longevity genes present a higher number of overlapping age-related diseases than anti-longevity genes for all orthologs (i.e., combining human orthologs of all genes from model organisms), mouse and *S. cerevisiae*. The opposite is verified for *Drosophila* and *C. elegans* sets (Fig. 2A).

Supplementary Materials, Tables S6 and S7 include the *P*-values and the number of CAD-genes for, respectively, age-related disease classes and individual diseases. Neoplasms (1.35E-56), nutritional and metabolic (9.70E-34), cardiovascular (2.00E-23) and nervous system (1.78E-18) classes have the strongest associations with human aging. There is an additional class not considered in the individual ARD analysis, the eye diseases, which presents a positive association with aging only for human aging-related genes and mouse pro-longevity genes (Supplementary Materials, Table S6).

#### Publication bias effects and correction

The inclusion of more and less studied genes may reduce the accuracy of the results. This is an issue when using large datasets that may contain systematic biases. Indeed, we observed a moderate correlation between the number of publications associated with a gene and its number of annotated protein-protein interactions (Spearman correlation coefficient = 0.67). While this is not unexpected, it could result in biases in systems biology analysis. To minimize this issue, a publication bias correction (PBC) based on the number of publications per gene was tested.

The first step of the PBC was setting a threshold for differentiation between more and less studied genes. Table 1 shows the average and the median number of publications computed for the following gene sets: human genome, human interactome, human aging-related genes and human homologs of aging-related genes from model organisms. The sets of aging-related genes have a higher average (range 13.0 to 30.3) and median (range 9 to 23) values when compared with the whole genome (average of 8.7 and median of 6) and the interactome (average of 10.4 and median of 8). This is expected but it shows that aging-related genes are more studied than average.

Thresholds between 8 and 20 publications were assessed in order to define the value with which the subsequent analyses were performed (see Supplementary Material, Fig. S2). Overall, we used 10 publications as a threshold.

#### Overlap between aging-related genes and age-related diseases with publication bias correction

The overlap analysis between aging-related genes and ARD genes was repeated after applying a filter for PBC (i.e., only containing genes with at least 10 publications). After PBC, the human aging-related gene set presents a significant overlap with ARD from all classes except for the immune system class (Fig. 2B). For the human homologs of aging-related genes from model organisms, only the mouse and the *Drosophila* present significant overlaps with ARDs and the latter only presents significant results for the anti-longevity gene set. In the mouse, pro-longevity genes have a higher number of ARDs overlapping compared to anti-longevity genes. In the all orthologs gene set, both anti- and pro-longevity genes show an association with the neoplasms class. These results are supported by *P*-values in Supplementary Materials, Tables S8 and S9, also suggesting a general stronger overlap with aging of genes associated with neoplasms and nutritional and metabolic diseases.

Comparing the two analyses without and with PBC, respectively, Figure 2A and B, we observe a decrease in the number of significant overlapping ARDs with all aging-related gene sets due to the exclusion of less studied genes. The human aging gene set is the least affected by the exclusion of less studied genes since after PBC it presents only a loss of 16% of its genes (298 to 253 genes). Small reductions are verified in small gene sets, such as the baker's yeast and the mouse. The opposite is verified in bigger gene sets, such as worms, which lose 47% and 50% of genes in anti- and pro-longevity sets, respectively. Finally, these same patterns are observed comparing Supplementary Materials, Tables S6–S9, which show statistical tests for the various overlaps.

#### Overlap between aging-related genes and age-related diseases in the interactome

The interactome (15000 genes) is a subset of the genome (20183 genes) within which only genes for which protein-protein interaction data is available are present (Table 1). We assessed the overlap between aging and ARDs genes when restricting the analysis to genes in the human interactome (Supplementary Material, Fig. S3). The distribution of aging-related gene overlaps with ARDs is similar in the interactome (Supplementary Material, Fig. S3) to the distribution in the whole genome (Fig. 2A), both without and with PBC. The analyses present similarities in the total number of genes, the overlap significance (*P*-values), the number of CAD-genes, and the relations between age-related diseases and aging shown by the anti- and pro-longevity sets.

When comparing the whole genome and the interactome analyses without PBC (Supplementary Materials, Tables S6, S7, S10 and S11) and the whole genome and the interactome analyses with PBC (Supplementary Materials, Tables S8, S9, S12 and S13), there is a slight drop in the significance of overlaps with PBC, suggesting that some (but not all) results are indeed due to publication bias. Looking at the effect of PBC on the number of CAD-genes, there is again a slight decrease with PBC in the majority of ARD classes and individual diseases. We conclude that publication bias has a modest but noticeable effect on our results.

**Figure 4.**
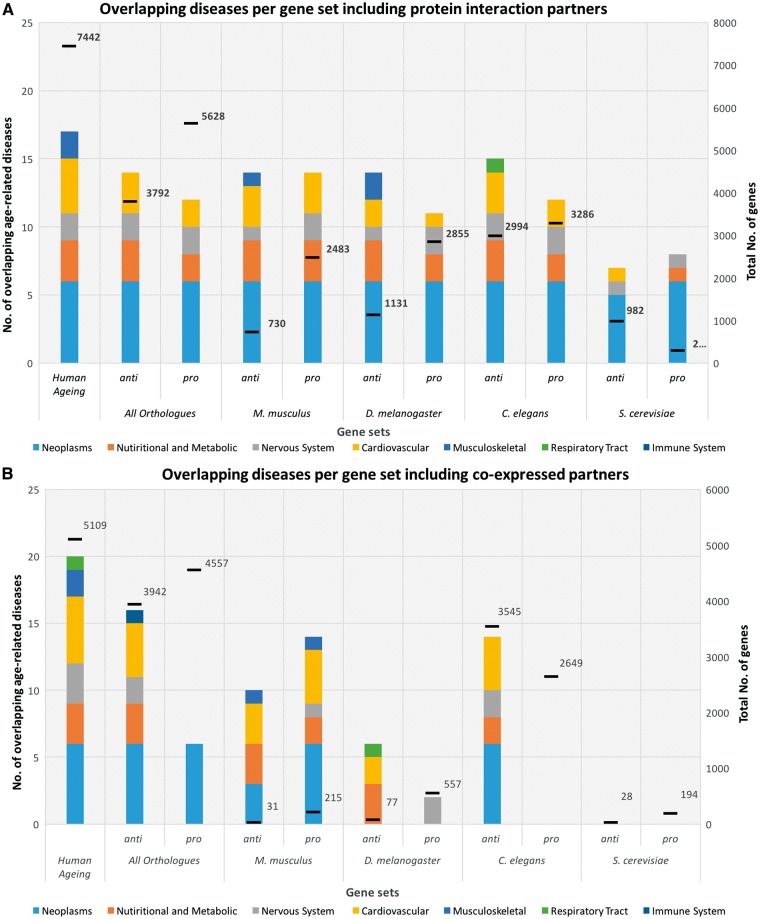
The y-axis quantifies the number of age-related diseases which significantly overlap with aging-related genes; the x-axis describes the aging-related gene sets studied according to the source organism (i.e., human plus human homologs of aging-related genes from each model organism). The columns have seven different colours to represent each age-related disease classe analysed: Neoplasms (light blue), Nutritional and Metabolic diseases (orange), Nervous System diseases (light grey), Cardiovascular diseases (yellow), Musculoskeletal diseases (blue), Respiratory Tract diseases (green) and Immune System diseases (dark blue). The first column represents the number of age-related diseases with a significant overlap with candidate human aging-associated genes. Model organisms are ordered by evolutionary proximity to humans. This analysis was performed with PBC. The secondary y-axis displays the number of genes from the respective gene sets. (**A**) shows the number of significant overlapping aging-related genes with age-related diseases, including first order interaction partners. The interactome plus aging-related and age-related disease genes was considered as background. (**B**) shows the number of significant overlapping aging-related genes with age-related diseases, including co-expressed genes. The genome was considered as background.

Since genes function in combination with other genes, studying genes and proteins as part of interaction networks is essential (21). To study the effect of genes which interact with aging-related genes in the links between aging and ARDs, we performed an analysis in which aging gene sets were composed of gene sets from the genome with PBC plus the genes directly connected to them (first order partners). This analysis revealed that aging-related gene sets including the first order partners are 18 to 51 times larger than the original aging-related gene sets with PBC (Fig. 4A). This increase in the number of genes is not proportional to the initial gene set size, and human aging-related genes are the ones that interact more with other genes.

Regarding ARDs classes overlapping with aging-related genes, neoplasms and nervous system classes do so in all gene sets analysed. Cardiovascular, as well nutritional and metabolic classes are also present. Musculoskeletal diseases overlap with human aging-related genes and then they only overlap with anti-longevity genes of mice and *Drosophila*. Finally, respiratory tract diseases present a significant overlap with aging for the anti-longevity gene set in *C. elegans*.

Overall, there is a clear increase in the number of overlapping ARD genes with aging-related genes by including first order interaction partners, as well as in the number of CAD-genes, which is supported by a statistical significance analysis (Supplementary Materials, Tables S14 and S15). Given the large increase in the number of genes by including first order partners, these results are not surprising but they underscore the large interconnection of biological networks, including between aging and age-related diseases.

#### Co-expression network analysis

Co-expression networks offer a complementary perspective on biological interactions from protein-protein interaction networks. To study co-expression, data were downloaded from the GeneFriends database (22) and genes co-expressed with aging-related genes (human candidate genes plus human homologs of genes associated with aging in model organisms, all following PBC) were considered (see Materials and Methods). The inclusion of genes co-expressed with aging-related genes again changes the number of genes per set; and there is an increase which is proportional to the size of the initial set, i.e., larger sets have a greater increase in their sizes (Fig. 4B).

**Figure 5.**
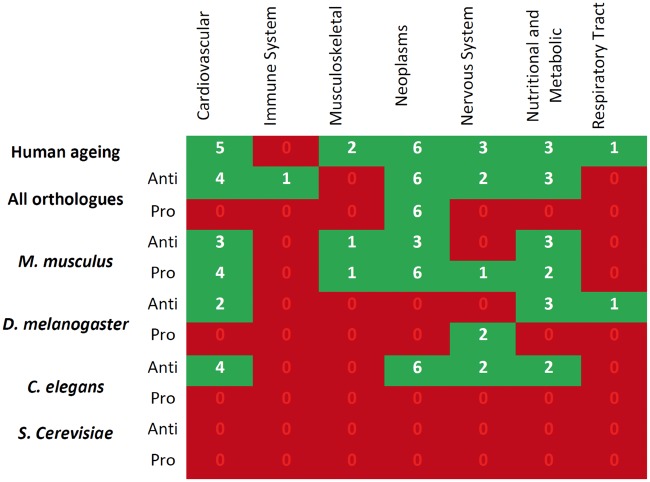
Overlapping aging-related genes and their co-expressed partners with age-related diseases for various classes and organisms. Green means there is at least one age-related disease from that class that significantly overlaps with aging-related genes and red means no association. Model organisms are in descending order of their proximity to humans. This analysis was performed without PBC.

At the level of age-related disease classes, neoplasms is the main class with a significant overlap between aging-related genes and ARDs genes, followed by the cardiovascular and nervous system classes. As before, the human aging-related gene set shows the highest association with ARDs. Immune system disease genes seem associated with aging-related genes when considering the anti-longevity genes of all orthologs together. Interestingly, there is a difference in overlapping classes between anti- and pro-longevity gene sets and, except in the mouse, anti-longevity genes present a higher relation to ARDs than pro-longevity genes, which is very clear in flies and worms (Fig. 5).

In the mouse, the results show that pro-longevity genes slightly overlap with nervous system disease genes, but the association is verified due to just one disease. In *Drosophila*, anti-longevity genes are associated with nutritional and metabolic, cardiovascular and respiratory tract diseases, while the pro-longevity genes are associated only with nervous system diseases. Finally, there is no significant overlap with any age-related diseases in the pro-longevity gene set of worms, even though it contains more than two thousand genes. Moreover, there are a few CAD-genes (up to 15) which show non-significant overlaps with the assessed ARDs (Supplementary Materials, Tables S16 and S17).

Because including co-expressed genes increases the number of starting genes, there is an increase in the number of CAD-genes when including co-expressed genes. Using the human aging-related gene set as an example, there are 65 CAD-genes in the overlap with neoplasms genes but when including the co-expressed genes the overlap with neoplasms increases to 131 CAD-genes. However, the percentage of overlapping genes drops dramatically. For human aging-related genes, 22% and 0.06% are associated with ARDs, with and without co-expressed genes, respectively.

### Properties of common genes between aging and age-related diseases

Common genes or CAD-genes from the overlap analyses can highlight clues about pathways which link aging to disease processes. CAD-genes were obtained from the overlap between the human aging set and ARDs genes from analyses per individual age-related diseases or per diseases class, both with PBC.

#### A small subset of aging-related genes are also associated with age-related diseases

**Figure 6.**
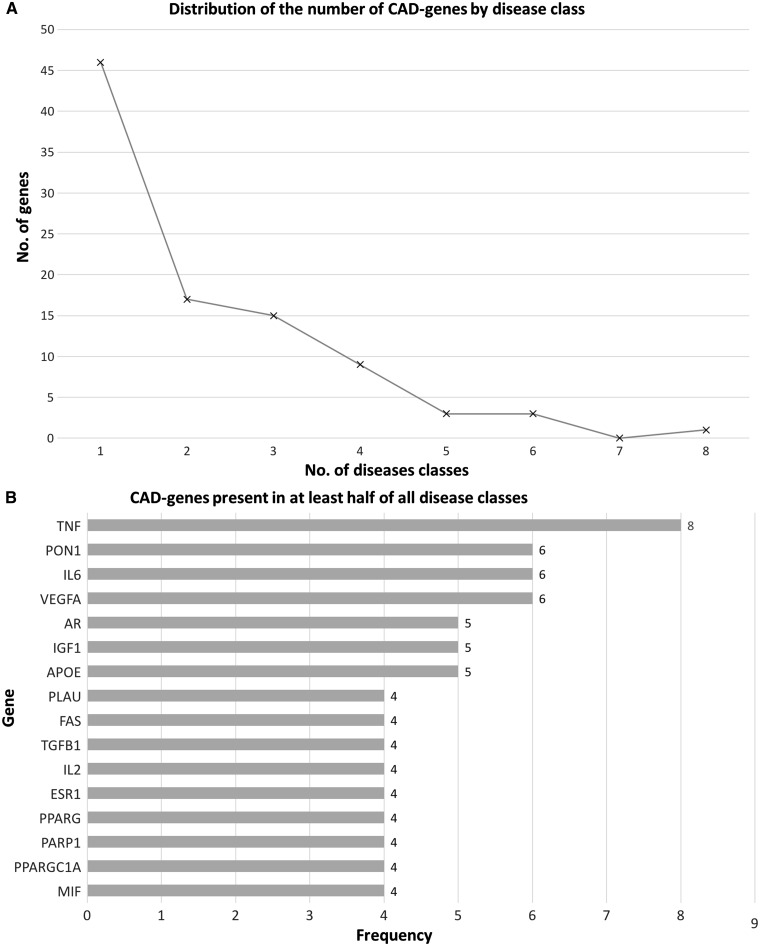
(**A**) CAD-genes distribution as associated with age-related disease classes. (**B**) shows the genes involved in half or more disease classes. *TNF* is associated with all the age-related disease classes analysed. This analysis was performed with PBC.

The number of times that each CAD-gene overlaps with ARDs was defined as its frequency, and allows us to determine if some genes are involved in several disease processes. Figure 6A shows the frequency of CAD-genes across all the age-related disease classes. A total of 94 genes were obtained from the overlap between the human aging-related genes and all the ARDs genes per class. A majority of these genes (approx. 83% genes) overlap with up to three classes (Fig. 6A). Regarding genes which overlap with a great number of classes, *PON1* and *APOE* are at the top (Fig. 6B), as well as some other genes involved in age-related changes, for example, *VEGFA*, *IL6* and *AR*. One gene present in all ARDs analysed is *TNF* (tumour necrosis factor).

It is also interesting to explore aging-related genes which are not associated with any ARDs. The 94 CAD genes represent 37% of the human aging-related genes with PBC (253 genes), which means that most (63%) aging-related genes are not associated with any ARD class. From the perspective of ARDs genes (639 genes), about 15% have been related to human aging.

**Figure 7.**
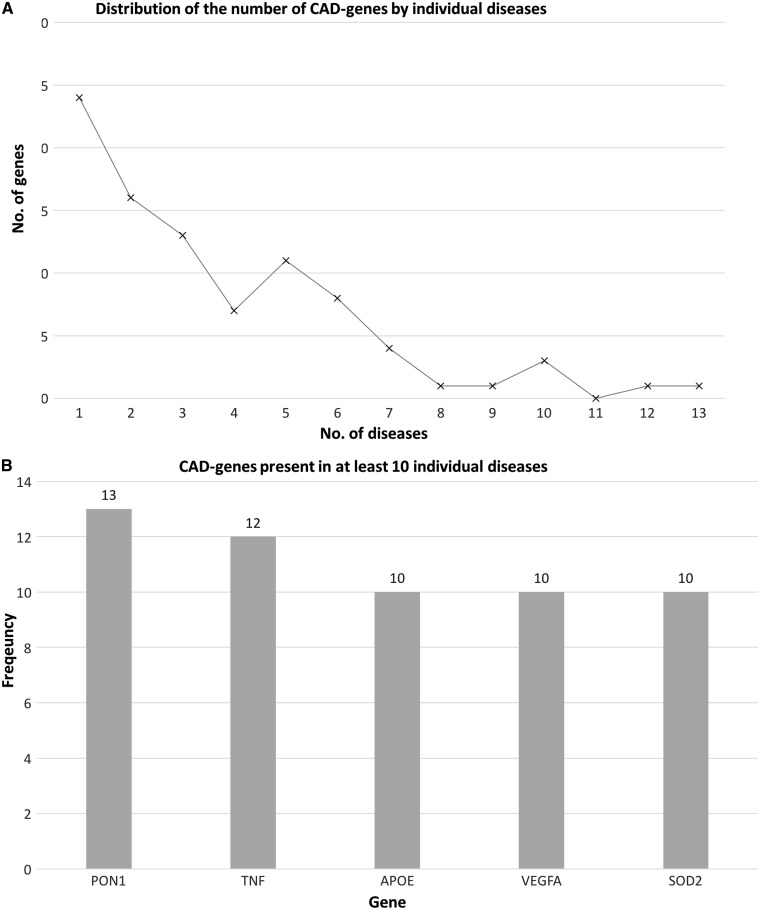
(**A**) CAD-gene distribution as associated with individual age-related diseases. (**B)** shows the CAD-genes involved in ten or more individual diseases, with *PON1*, *TNF*, *APOE* the top 3 genes associated with the greatest number of age-related diseases. This analysis was performed with PBC.

An analysis of the CAD-genes distribution was also performed by individual ARDs. A total of 90 genes were found to overlap between human aging-related genes and ARD genes. Figure 7A shows that the number of genes involved in several ARDs is small, and about 59% (53 genes) of the 90 genes are associated with up to three ARDs. The pattern of distribution is similar to the analysis by ARD class and *TNF*, *PON1*, *APOE* and *VEGFA* are the top of CAD-genes among ARDs for both analyses (per age-related disease class and per individual disease) (Fig. 7B). In this analysis, the percentage of aging-related genes not associated with any age-related disease is about 64%. Similar to above, from the perspective of ARD genes (596 genes), only 15% have been related to human aging.

#### Pathways and processes linking aging and age-related diseases

A functional enrichment analysis was performed on CAD-genes. The background used was the set of human aging-related genes plus ARDs sets. Functional enrichment for CAD-genes from all analysed ARD classes shows that these genes are associated with: negative regulation of apoptosis, cell cycle, positive regulation of DNA, positive regulation of protein metabolic process and response to stimuli (Supplementary Material, Table S1).

Looking at CAD-genes in individual ARDs, only neoplasms, nutritional and metabolic, musculoskeletal and eye classes have significant functional clusters. Relative to neoplasms, CAD-genes are more associated with negative regulation of apoptosis, DNA repair, regulation of cell cycle and cancer, which is in line with cancer aetiology and its relationship to aging (17). CAD-genes from the nutritional and metabolic class are related to response to insulin stimulus and positive regulation of lipid process, while CAD-genes for musculoskeletal diseases only show an association with the extracellular region. Finally, eye diseases CAD-genes seem to be associated with positive regulation of RNA metabolic process (Supplementary Material, Table S1). 

#### Increased network connectivity in genes common to aging and age-related diseases

Network approaches consider as a measure of node (gene) relevance the node's degree, which represents the number of connections of each node. This measure helps to define hubs, which in general are deemed essential nodes with many connections. To understand if CAD-genes are likely to be hubs, a comparison between the degree of CAD-genes and ARD genes or aging-related genes (the non-common genes) was made using protein-protein interaction data (Supplementary Material, Table S2).

Age-related disease class analysis shows significant differences in node degree between CAD-genes and controls (*P*-value < 0.001). The median node degree of CAD-genes (47) is substantially higher than the median for the control set (11). Looking at ARD classes, only two classes have a significant (*P* < 0.05) difference between the two sets investigated: neoplasms and immune system diseases. Neoplasms present a higher median for CAD-genes (47) compared to the control set (23.5), while for the immune system class the opposite is verified (8.5 vs 43) (Supplementary Material, Table S2).

The results from the analysis per individual ARDs show a significant difference in the number of node connections for four diseases: atherosclerosis (*P =* 0.002), breast neoplasm (*P =* 0.020), hypersensitivity (*P =* 0.019) and osteoporosis (*P =* 0.039). Except for breast neoplasm, the median for CAD-genes is lower when compared to the ARD genes (Supplementary Material, Table S2).

#### Processes associated with aging-related genes not associated with age-related diseases

A functional enrichment was performed for genes from the human aging gene set which are not associated with any ARD. The main processes in the functional enrichment are: response to DNA damage, negative regulation of apoptosis, ATP-binding, negative regulation of transcription, DNA repair, aging, telomere maintenance, response to several stimuli, negative regulation of gene expression, cancer and signalling pathways (for examples, insulin, IL3 and MAPKinase). Of these terms, the ones with the higher cluster scores are response to DNA damage and negative regulation of apoptosis. The full list of significantly enriched terms is in Supplementary Material, Table S3.

### Molecular evolutionary rates of aging- and disease-related genes

Aging-related and disease-related genes are also known to differ from the genome-wide average at the level of selection pressures. The study this, the dN/dS ratio between humans and mice of the human aging-related genes and the ARD genes sets was analysed and compared to the remaining genome (see Materials and Methods). Results show a significant (p < 0.001) difference between ARD genes and the other genes in the genome, wherein ARD genes have a higher median dN/dS ratio (0.137) than the whole genome (0.091). Although there was a difference between aging (median of 0.079) and non-aging genes (median of 0.093), this was not statistically significant (p-value = 0.155).

The dN/dS ratio was also assessed in anti- and pro-longevity genes. A difference in dN/dS ratio between anti- and pro-longevity genes was only observed in *C. elegans* (p-value = 0.046, which is not significant after Bonferroni correction), so we find no evidence of differences in molecular evolution rates between anti- and pro-longevity genes.

Searching for patterns and features which could define CAD-genes, their molecular evolutionary (dN/dS) rate was analysed in comparison with aging-related genes and ARD genes. The CAD-genes used were from the overlaps of the three ARD classes with more genes: all classes together, neoplasms and nutritional and metabolic diseases. No statistically significant differences were found, suggesting that molecular evolution rates of CAD-genes are not different from other aging and ARD genes.

### Drugs predicted from aging-related gene interactions with drugs

Given the large number of aging-related genes and pathways identified, there is great interest in identifying drugs that target them and may potentially have clinical benefits (23). To obtain candidate drugs affecting the aging process, we employed publicly available drug-gene interaction data (see Materials and Methods). In total, 376 drugs whose targets overlapped with aging-related genes were obtained. Twenty statistically-significant drugs that have more interactions with aging-related genes than expected by chance were obtained after Bonferroni correction (Supplementary Material, Table S4).

The majority of the drugs obtained from this analysis were histone deacetylase inhibitors used for the treatment of cancer. This might be due to an overrepresentation of cancer drugs in public databases. Nonetheless, three known lifespan-extending drugs were identified: sodium phenylbutyrate, valproic acid and everolimus (Supplementary Material, Table S4). The fact that experimentally validated aging-related drugs are detected by our methodology suggests that this approach may be useful to identify new candidate drugs with effects on aging.

## Discussion

To our knowledge, ours is the largest analysis of the gerontome to date, and the first to consider pro- and anti-longevity genes in a systematic fashion. We first characterized functions and pathways overrepresented in pro- and anti-longevity genes. Major anti-longevity pathways and processes include insulin signalling, growth hormone signalling and mTOR signalling. Key pro-longevity pathways include p53, cell cycle and autophagy. Although such pathways and processes are known to be related to aging (2,4,5,24), it is interesting that they are classified as anti-and pro-longevity in our systematic analysis of the genetics of aging. Differentiation between anti-longevity and pro-longevity genes and processes can provide additional clues about aging-related processes and can help identify other genes with a similar effect on aging.

In order to find relations between aging and ARDs, we compared aging-related gene sets with ARD genes. Limitations of our study include the fact that possibly many genes associated with longevity and diseases remain to be identified, and the causal genes in many genetic associations with disease are still unknown. In spite of these caveats, our results show an association between aging and ARDs at the genetic level, although this is surprisingly species-specific with a stronger overlap in mice than in invertebrates (flies and worms) and practically no overlap in yeast.

**Figure 8.**
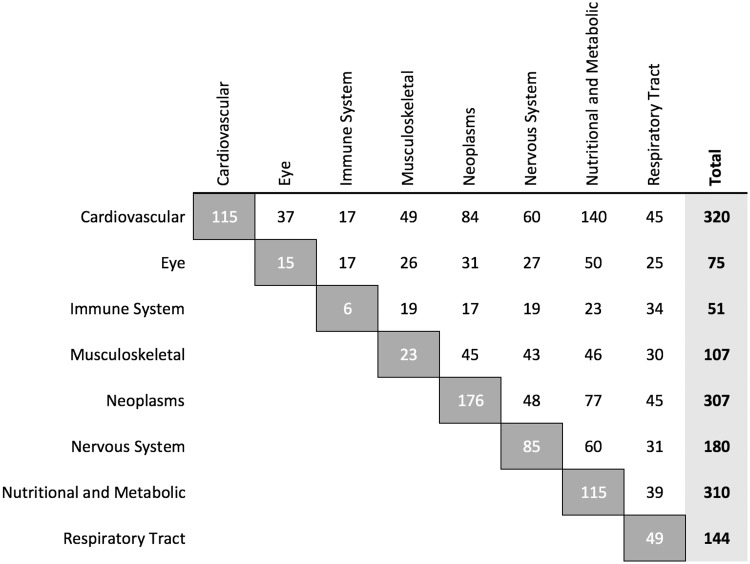
Number of genes by age-related disease class (Total column) and shared with each other disease classes. The white cells present the number of genes shared between disease classes and the darker grey cells show the number of genes not shared with any other disease class.

The overlap analyses of anti- and pro-longevity genes shows differences in musculoskeletal, nervous system and cardiovascular diseases. The identified overlaps suggest that the musculoskeletal and nervous systems are related to pro-longevity genes while anti-longevity genes seem more associated with cardiovascular diseases. Looking at ARD classes which overlap with human aging-related genes, a significant overlap is verified for all classes as expected, except for immune system diseases in the analysis with PBC. The nutritional and metabolic diseases, the neoplasms, the cardiovascular diseases and the nervous system diseases have the most significant overlap with human aging-related genes. Eye diseases, respiratory tract diseases (which we considered a negative control) and immune system diseases had the least overlap, but it is important to mention that these are (together with musculoskeletal diseases) the age-related disease classes with fewer genes (Fig. 8).

Genes historically associated with diseases are more likely to be studied. A publication bias correction approach, based on the number of publications associated with each gene, was applied in order to explore and reduce such biases. The analyses with and without PBC, when compared, show the effect of the removal of less studied genes (Fig. 2A vs. B). The overlaps for *C. elegans* and *S. cerevisiae* disappear when the PBC is applied, which supports the hypothesis that some overlaps are statistically significant only due to an overrepresentation of better-studied genes. The comparison of analyses with and without PBC proves that systematic researcher biases can influence the results in large-scale systems biology, genomic and genetic analysis.

From our network analysis including the first order protein-protein interaction partners, it is possible to conclude that aging-related genes are widely connected to other genes, which is supported by the huge increase in gene sets’ sizes (Fig. 4A). There is an increase in the number of CAD-genes (the common or shared genes by aging and ARD) when including the first order partners, which suggests widespread interactions between aging-related genes and genes associated with age-related diseases. The results are also in agreement with recent research using genome-wide association studies (GWAS) data, which showed the same conclusion for five age-related categories: neurodegenerative, cancers, cardiovascular, metabolic and other diseases (25). A co-expression analysis of the links between aging and ARDs supports the idea of species-specific effects, but with more anti-longevity genes in invertebrates being related to ARDs. It is tempting to speculate that perhaps anti-longevity genes work together more tightly in transcriptional networks than pro-longevity genes.

Previous studies of the association between aging and diseases have demonstrated that the association is established by a small number of genes (25). Indeed, in the present analysis, CAD-genes represent a minority of the aging-related genes. CAD-genes are mainly related to apoptosis, metabolic regulation and DNA damage. These processes are similar to those previously reported to be associated with aging and may hint at underlying mechanisms important in various age-related diseases. CAD-genes also showed a higher number of connections with other genes than the remaining genome, which suggests that those genes tend to be hubs in networks. *TNF*, *PON1*, *APOE* and *VEGFA* are present in a great number of ARDs, which is in line with their involvement in some of the essential pathways whose disruption compromises metabolism and can lead to pathologies (26,27).

The dN/dS ratio analysis showed a statistically significant higher dN/dS ratio of ARD genes when compared to the remaining genome, while aging-related genes had a lower dN/dS ratio that was not statistically significant. Therefore, we can affirm that ARD genes have a higher predisposition to changes in their sequence than aging-related genes. These results are in line with previous findings: an analysis using a previous version of GenAge found that aging-related genes have a lower dN/dS ratio (28). One previous study found a higher molecular evolutionary rate in disease genes (29). Our results further suggest that aging-related genes tend to be evolutionarily conserved, perhaps because they are part of essential pathways and conserved pleiotropic effects on aging (28), while genes associated with age-related diseases may be under relaxed selection given that they impact later in life.

Finally, taking advantage of a database of gene-drug interactions, we mapped GenAge’s genes to drugs and obtained a list of 20 candidate drugs for aging effects. Of these, three are already experimentally validated and the rest is yet to be explored. As such, these compounds are promising for future studies.

## Concluding Remarks

The main conclusion from this work is that aging and age-related diseases are related and share more genes than expected by chance. Human aging-related genes showed a considerable overlap with ARDs. These overlaps are driven by a small subset of aging-related genes which are associated with various age-related diseases and are hubs in networks. Besides, the extent of overlaps decreases with evolutionary distance, and yeast aging-related genes show practically no overlap with ARDs. Novel differences in overlapping age-related disease classes between anti- and pro-longevity genes were observed: Nervous system and musculoskeletal diseases seem more associated with pro-longevity, while cardiovascular diseases have a stronger association with anti-longevity genes. Moreover, network analyses (protein-protein interactions (PPI) and co-expression) suggest the existence of intermediate genes which promote the associations between aging and age-related disease genes. Overall, our work establishes a new standard in the analysis of aging-related genes in a systematic way.

## Materials and Methods

### Aging- and longevity-associated genes

Aging-associated genes were obtained from GenAge Build 17 (6). These include 298 human candidate aging-related genes. GenAge also includes aging- or longevity-related genes in model organisms. For use with human datasets, human orthologs of model organism genes were used, composed of 1037 genes from the four main biomedical model organisms: mouse, fruit fly, roundworm and baker's yeast. The genes of each model organism were separated by their longevity classification: anti- or pro-longevity. Pro-longevity genes are genes whose decreased expression (due to knockout, mutations or RNA interference) reduces lifespan and/or whose overexpression extends lifespan; conversely, anti-longevity genes are those whose decreased expression extends lifespan and/or whose overexpression decreases it (6). Genes which were not included in one of these two longevity classes were excluded. A small number (19) of genes with both anti- and pro-longevity classifications were also excluded.

To sum up the data, aging-related genes were divided into 11 gene sets: one set of 298 human aging-related genes, two sets (anti- and pro-longevity) of human orthologs from each model organism and two sets with all human orthologs of genes in all model organisms. The mouse sets (i.e. human orthologs of genes associated with aging in mice), have 23 and 59 genes, the fruit fly sets have 48 and 87 genes, the roundworm sets have 381 and 290 genes, and lastly the baker's yeast sets have 41 and 13 genes, respectively anti- and pro-longevity (Supplementary Materials, Table S18). Finally, sets with all orthologs have 448 and 421 genes, anti- and pro-longevity sets, respectively. The full lists of human aging-related genes and human orthologs are available in the supplementary material (Supplementary Dataset 4).

Data on human genes associated with longevity in genetic association studies were obtained from the LongevityMap build 1 (20). In the full set of 755 genes, there were 328 genes with at least one significant result reported.

### Age-related disease genes

Age-related disease (ARDs) genes were assembled on 15-04-2015 from a diseases list compiled by a National Institute of Aging study. The list only includes genes with an association with the disease phenotype and with a MeSH annotation (30). This list is available online (https://www.irp.nia.nih.gov/branches/rrb/dna/gene_sets.htm) and it was compiled using information from the Genetic Association Database (30).

The original list includes many diseases not relevant for the present analysis since our interest focuses on complex ARDs. To select relevant ARDs, diseases with fewer than 20 genes associated and diseases of non-age-related disease classes were excluded. We chose a threshold of 20 genes because it captures the major age-related diseases yet not so many diseases that our findings end up being diluted (Supplementary Material, Table S19). The original list also includes processes and conditions, for example, insulin resistance and hyperlipidemia, which are dysfunctions, and for that reason were also excluded. The following analysed classes were described as age-related in the literature: cardiovascular diseases, eye diseases, immune system diseases, musculoskeletal diseases, nervous system diseases, nutritional and metabolic diseases and neoplasms (2,31). Respiratory tract diseases were considered as negative controls since the two diseases (after application of the described selection criteria) in this class are asthma, which is not considered an age-related disease (32), and chronic obstruction pulmonary disease, which is primarily environmental.

Selection of individually studied ARDs was made based on two criteria: first, the number of genes, to have larger sample sizes and increase statistical power. The second criterion was how often and common was each disease. An example of selection is the case of ovarian neoplasm, which presents a smaller number of genes and is better known than head and neck neoplasms. In order to have a representative selection, seven diseases classes were included in the individual age-related disease analysis; for classes with a large number of diseases, we selected the top five or six most representative individual diseases of the class. Eye diseases were excluded from the individual disease analysis, since they include only non-common diseases with a small number of genes. Diseases that are primarily driven by environmental factors, like chronic obstruction pulmonary disease, were also not studied.

In total, 893 different genes associated with ARDs were considered. Figure 8 shows the number of genes per ARD class and the number of genes shared with each one of the remaining classes. The list of ARDs used in the present analysis is summarized in Supplementary Material, Table S20. In total, 40 diseases were part of ARD classes, of which 22 ARDs were analysed individually. The list of all age-related diseases and their related genes is available in the supplementary material (Supplementary Dataset 5). Our full datasets are also available on GitHub (https://github.com/maglab/genage-analysis).

### Protein–protein interaction and gene co-expression data

Protein–protein interactions were obtained using the BioGRID plug-in available in Cytoscape, on 16-04-2015, by downloading the available node and edge tables. The two main types of interactions (‘physical association’ and ‘direct interaction’) represent 124,238 of 140,891 interactions, involving 14,721 of 15,000 proteins. As such, the interactome analysis was performed using the full interactome. To obtain the first-order partners of aging-related genes, a Python script was used to compile connections between all genes in the interactome and return the merged list of seed genes and genes connected to them.

Co-expression data from RNA-Seq was obtained using GeneFriends (22) on 03-06-2015. To obtain the co-expressed genes, a significance threshold of 2.5E-06 was applied to the p-value retrieved from GeneFriends. The threshold was defined by correction of standard α (0.05) using a Bonferroni correction where *N* represents the genome size (20183 genes).

### Publication bias correction (PBC)

The number of publications was compiled from the Swiss-Prot PubMed annotation list, downloaded on 23-04-2015. Only human and reviewed genes were considered for this analysis. Although PubMed publications annotated in Swiss-Prot are not the total number of publications for each entry, they represent a curated selection. Thus, Swiss-Prot was selected as the source for the number of publications due to its curated nature, which makes it a reliable source of annotated data for protein coding genes. 

### Gene features

The list of all human genes was collected from GenBank on 31-01-2015. For this analysis, only human annotated and protein-coding genes were considered, which represent a set of 20,183 genes.

Molecular evolutionary rate (dN/dS) was calculated from the number of synonymous (dN) and non-synonymous (dS) substitutions downloaded from Ensembl BioMart, selecting the Ensembl Genes 80 database and *Homo sapiens* genes (GRCh38.p2) dataset on 17-05-2015. Of all the model organisms considered in the present work, only mouse orthologs present dN and dS values, due to the great evolutionary distance shown by the other organisms. Thus, all dN/dS ratio comparisons consider the evolution between mice and humans.

### Feature selection method

We used a recently proposed feature selection method, from the area of data mining (or machine learning) to select the relevant Gene Ontology (GO) terms for predicting the pro-longevity or anti-longevity effect of a gene on a model organism (8). In essence, we addressed the classification task of data mining, where the goal is to predict the class (pro- or anti-longevity effect) of an instance (a gene) based on predictive features (GO terms) associated with that gene. The used feature selection method differs from other feature selection methods typically used in data mining in two important ways, as follows. First, it selects a specific set of features relevant for the classification of each instance, instead of selecting the same set of features for all instances, as usual in data mining. This increases the flexibility of the feature selection process, recognizing that the optimal set of GO terms for predicting the pro- or anti-longevity effect of a gene varies across different genes. Second, the used method performs ‘hierarchical’ feature selection, in the sense that it takes into account the hierarchical structure of the GO in order to improve the feature selection process; unlike conventional (‘flat’) feature selection methods.

That feature selection method was applied to datasets with data about aging-related genes from the four traditional model organisms, namely: mouse, fly, roundworm and yeast. The results of the feature selection method were transformed into a rank of GO terms as follows. For each dataset (model organism), for each GO term, we counted the number of instances (genes) where that GO term was one of the relevant features selected by the feature selection method for predicting the pro- or anti-longevity effect of the gene. Then, we ranked the GO terms in decreasing order of this frequency of selection. We also used a statistical test of significance based on the binomial distribution to detect which GO terms were significantly associated with the class being predicted. A detailed description of the feature selection method and how its results were used to rank the GO terms can be found in (8).

### Overlap analysis

A significant overlap between aging-related genes and ARDs is defined as: i) an observed number of CAD-genes above the number of CAD-genes expected by chance; and ii) a p-value below 0.05 (Fisher's exact test). Genome and interactome analysis used whole genome and interactome as background, respectively. The background for the first order partners analysis was the seed list plus the interactome and was adjusted for each aging gene set since the seed list varies between different aging sets. In this analysis, the interactome was added to the background since the first order partners were selected from that group.

### Functional enrichment analysis

Functional enrichment analysis using the Database of Annotation Visualization and Integrated Discovery (DAVID) (33) was performed to identify overrepresented categories. The analysis was done by running the Functional Annotation Clustering module under default parameters. Unless otherwise stated, the whole genome was used as background. Enrichment scores (E. Score) above 1.3 (which corresponds to *P =* 0.05) are widely accepted as relevant (33); however, in this analysis a threshold of 2.5 (corresponding to *P =* 0.003) was used for more significant results. A Benjamini correction was applied for correcting for multiple hypothesis testing.

### Candidate drugs from GenAge targets

To identify candidate drugs with possible anti-aging properties, the Drug Gene Interaction Database (DGIdb) version 2 (34) was used. We classified all 44 types of drug-gene interactions in DGIDB into either ‘Anti’ (decrease gene expression or activity) or ‘Pro’ (increase gene expression or activity) or ‘Neither’ (non-applicable or undefined effects), so that they can be matched with GenAge genes to obtain a putative lifespan-extending effect (Supplementary Material, Table S5). Drugs were obtained by considering if they interact with GenAge genes in a way that would be predicted to extend lifespan. That is, for an ‘Anti’ drug, only the interactions with anti-longevity genes are scored; and vice-versa for ‘Pro’ drugs.

In total, 376 drugs were obtained which were ordered based on ascending *P*-value obtained using a one-tailed hypergeometric test. After Bonferroni correction (α = 0.05), 20 statistically significant drugs were obtained.

## Supplementary Material

Supplementary Material is available at *HMG* online.

## Supplementary Material

Supplementary Data
