# Supplementary material for: Systematic analysis of the gerontome reveals links between aging and age-related diseases
Source: Hum Mol Genet. 2016 Sep 4;25(21):4804–18. doi: 10.1093/hmg/ddw307 (PMC5418736; doi:10.1093/hmg/ddw307)
Supplement: Supplementary Data [file ddw307_Supp.zip › ddw307-suppl_data/Supplementary_material.pdf]

Systematic analysis of the gerontome reveals links between aging and age-related diseases

Fernandes et al.

Supplementary Material

List of Supplementary Tables

Table S1 - Functional annotation of CAD-genes - aging or diseases genes
Table S2 - Number of connections analysis for CAD-genes - per class and disease
Table S3 - Functional annotation of non-overlapping genes – genome
Table S4 - List of candidate drugs for lifespan extension. Drugs colored green are experimentally validated
Table S5 - Categorization of DGIdb's drug-gene interactions into Anti/Pro/Neither classes
Table S6- Overlap analysis per class - Genome
Table S7- Overlap analysis per disease - Genome
Table S8- Overlap analysis per class - Genome with Publication Bias Correction
Table S9- Overlap analysis per disease - Genome with Publication Bias Correction
Table S10- Overlap analysis per class - Interactome
Table S11- Overlap analysis per disease - Interactome
Table S12- Overlap analysis per class - Interactome with Publication Bias Correction
Table S13- Overlap analysis per disease - Interactome with Publication Bias Correction
Table S14- Overlap analysis per class - including first order partners
Table S15- Overlap analysis per disease - including first order partners
Table S16- Overlap analysis per class - including co-expressed genes
Table S17- Overlap analysis per disease - including co-expressed genes
Table S18 - Number of genes of aging gene sets
Table S19 - Number of diseases at different thresholds for minimum number of genes
Table S20 - Diseases list with the disease class and the number of genes

List of Supplementary Figures

Figure S1: Similar top functional annotation clusters from the LongevityMap in two backgrounds
Figure S2: Analysis of publications number per gene for the human genome
Figure S3: Number of significant diseases per gene set - Interactome without and with PBC.

List of Supplementary Datasets (available at: https://github.com/maglab/genage-analysis)

Dataset 1: Functional enrichment of pro- and anti-longevity genes for each model organism
Dataset 2: Ranking of GO terms for each model organism according to selection frequency by a machine learning method
Dataset 3: Functional enrichment of human longevity-associated genes from the LongevityMap
Dataset 4: Full list of human aging-related genes and human orthologs from model organisms
Dataset 5: Full list of disease-related genes

Supplementary References

Supplementary Tables

Table S1 - Functional annotation of CAD-genes - aging or diseases genes

Terms Categories	Terms Summary	E. Score
**Aging / All diseases classes**		
GOTERM_BP_FAT	Negative regulation of apoptosis, cell death.	9.29
GOTERM_BP_FAT	Positive regulation of apoptosis, cell death, DNA damage response.	4.74
GOTERM_CC_FAT<br>UP_SEQ_FEATURE<br>GOTERM_MF_FAT<br>SP_PIR_KEYWORDS	Lumen, DNA binding, nucleus.	4.54
GOTERM_BP_FAT<br>SP_PIR_KEYWORDS	Cell Cycle.	4.25
GOTERM_BP_FAT<br>GOTERM_CC_FAT<br>SP_PIR_KEYWORDS	DNA repair, DNA damage, response to stress, nucleoplasm.	4.24
GOTERM_BP_FAT	Response to stimulus, response to hormones.	4.03
KEGG_PATHWAYS	Cancer.	3.84
GOTERM_BP_FAT	Response to UV radiation.	3.78
GOTERM_BP_FAT<br>GOTERM_MF_FAT<br>SP_PIR_KEYWORDS	Positive regulation of biosynthetic process, positive regulation of metabolic process, regulation of transcription.	3.60
GOTERM_BP_FAT	Positive regulation of DNA metabolic process, positive regulation of DNA replication.	3.12
GOTERM_BP_FAT	Positive regulation of protein metabolic process, positive regulation of phosphorylation, positive regulation of kinases cascade.	3.11
GOTERM_BP_FAT	& Positive regulation of cell motion, positive regulation of carbohydrates.	2.99
GOTERM_BP_FAT	DNA catabolic process.	2.96
GOTERM_BP_FAT	Response to hormonal stimulus.	2.91
GOTERM_BP_FAT	Apoptosis, cell death.	2.89
GOTERM_BP_FAT	Response to oxidative stress	2.56
GOTERM_BP_FAT	Protein complex.	2.55
GOTERM_BP_FAT	Neuronal development, neuronal differentiation	2.52
**Aging / Neoplasms**		
GOTERM_BP_FAT	Negative regulation of apoptosis, cell death.	5.80
SP_PIR_KEYWORDS<br>GOTERM_BP_FAT<br>GOTERM_MF_FAT<br>GOTERM_CC_FAT	DNA repair, DNA damage, DNA binding, nucleoplasm.	4.38
GOTERM_BP_FAT	Regulation of cell cycle.	3.93
SP_PIR_KEYWORDS<br>GOTERM_BP_FAT	Tumor suppressor.	3.51
KEGG_PATHWAYS	Cancer.	3.20
GOTERM_CC_FAT	Nucleoplasm.	2.60

**Aging / Nutritional and Metabolic diseases**		
GOTERM_BP_FAT	Response to insulin stimulus.	3.45
GOTERM_BP_FAT	Positive regulation of lipid metabolic process.	2.51
**Aging / Musculoskeletal diseases**		
SP_PIR_KEYWORDS<br>GOTERM_CC_FAT	Secreted, extracellular region.	3.10
**Aging / Eye diseases**		
GOTERM_BP_FAT	Positive regulation of RNA metabolic process.	2.93

E.Score - Enrichment Score

Table S2 - Number of connections analysis for CAD-genes - per class and disease

Class/Disease	Median of CAD-genes	Median of Aging or Disease Genes	MW
	**Per Class**		
**All Classes**	**47**	11	**<0.001**
**Cardiovascular**	29	17	0.438
**Eye**	47	37	0.606
**Immune System**	8.5	**43**	**0.047**
**Musculoskeletal**	14.5	37	0.107
**Neoplasms**	**47**	23.5	**0.001**
**Nervous System**	45	30	0.398
**Nutritional and Metabolic diseases**	30.5	19	0.324
**Respiratory Tract**	39	32	0.136
	**Per Disease**		
**Adenocarcinoma**	71.5	40	0.183
**Alzheimer's Disease**	46	36	0.324
**Arteriosclerosis**	22.5	44.5	0.123
**Arthritis**	29.5	38.5	0.949
**Asthma**	27	34	0.519
**Atherosclerosis**	3	**46**	**0.002**
**Autoimmune disease**	39	47	0.847
**Breast Neoplasm**	**49**	37	**0.020**
**Colorectal Neoplasm**	50.5	40	0.520
**Coronary Disease**	35.5	32	0.805
**Diabetes Mellitus, type 1**	12.5	37	0.106
**Diabetes Mellitus, type 2**	29	25	0.908
**Hypersensitivity**	2	**46**	**0.019**
**Hypertension**	28	27	0.231
**Lung Neoplasm**	47	37	0.354
**Multiple Sclerosis**	43	45	0.340
**Myocardial Infarction**	27	30	0.720
**Obesity**	35.5	37	0.375
**Osteoporosis**	6	**47**	**0.039**
**Parkinson's Disease**	34.5	45	0.227
**Prostatic Neoplasm**	27	42	0.847
**Stomach Neoplasm**	27	42	0.833

MW - p-value from Mann-Whitney U test

Table S3 - Functional annotation of non-overlapping genes - genome

Terms Categories	Terms Summary	E. Score
**Aging / All diseases classes**		
GOTERM_BP_FAT	Response to DNA damage stimulus, DNA metabolic process, DNA repair.	22.70
GOTERM_CC_FAT<br>SP_PIR_KEYWORDS<br>GOTERM_MF_FAT<br>GOTERM_BP_FAT	Nucleus, lumen, regulation of transcription.	19.37
GOTERM_BP_FAT<br>SP_PIR_KEYWORDS	Negative regulation of apoptosis, positive regulation of apoptosis, cell death.	14.02
GOTERM_BP_FAT<br>SP_PIR_KEYWORDS<br>GOTERM_MF_FAT<br>GOTERM_CC_FAT	Positive regulation of biosynthetic process, positive regulation of gene expression, positive regulation of transcription, DNA binding.	13.91
GOTERM_BP_FAT	Activation of receptor protein signalling pathway.	10.06
GOTERM_MF_FAT	Protein dimerization.	9.56
GOTERM_BP_FAT	Response to radiation, UV.	9.45
SP_PIR_KEYWORDS<br>GOTERM_BP_FAT<br>UP_SEQ_FEATURE<br>INTERPRO<br>GOTERM_MF_FAT<br>SMART	ATP-binding, protein phosphorylation, Serine/threonine protein kinase.	8.18
GOTERM_BP_FAT<br>BIOCARTA	Response to hormone stimulus, response to insulin stimulus, PTEN dependent cell cycle arrest and apoptosis.	7.96
GOTERM_BP_FAT<br>GOTERM_MF_FAT	Negative regulation of cellular biosynthetic process, negative regulation of transcription.	7.73
GOTERM_CC_FAT	Nuclear chromatin.	7.70
GOTERM_BP_FAT	Positive regulation of multicellular organism growth.	5.53
KEGG_PATHWAYS<br>UP_SEQ_FEATURE<br>INTERPRO<br>GOTERM_MF_FAT	Insulin signalling pathway, cancer, type II Diabetes Mellitus, MAP kinase activity, apoptosis.	5.33
GOTERM_BP_FAT<br>SP_PIR_KEYWORDS	DNA repair, DNA recombination, DNA damage.	5.33
GOTERM_BP_FAT	Cell cycle checkpoint.	5.29
GOTERM_BP_FAT	Response to oxidative stress.	5.22
GOTERM_MF_FAT	Transcription cofactor activity.	5.04
GOTERM_BP_FAT	Aging, response to extracellular stimulus.	4.97
GOTERM_BP_FAT<br>SP_PIR_KEYWORDS	Regulation of mitotic cell cycle, interphase.	4.81
GOTERM_CC_FAT<br>GOTERM_MF_FAT<br>GOTERM_BP_FAT<br>SP_PIR_KEYWORDS<br>KEGG_PATHWAYS<br>BIOCARTA	Telomere maintenance, negative regulation of DNA replication, positive regulation of DNA metabolic process.	4.60

GOTERM_BP_FAT	Positive regulation of protein kinase activity, positive regulation of transferase activity, MAPKKK cascade.	4.40
GOTERM_BP_FAT	Transcription.	4.38
GOTERM_BP_FAT	Response to drug, response to bacterium, response to mechanical stimulus.	3.70
GOTERM_BP_FAT	Regulation of cytokine production.	3.58
GOTERM_BP_FAT<br>GOTERM_MF_FAT<br>INTERPRO<br>GOTERM_CC_FAT<br>PIR_SUPERFAMILY<br>COG_ONTOLOGY	Negative regulation of gene expression, histone deacetylase activity, chromatin modification, negative regulation of cell cycle.	3.57
GOTERM_BP_FAT	Regulation of protein binding	3.49
UP_SEQ_FEATURE<br>INTERPRO<br>SMART<br>GOTERM_MF_FAT<br>GOTERM_BP_FAT<br>BIOCARTA	Transmembrane receptor protein serine/threonine kinase signaling pathway.	3.40
GOTERM_BP_FAT<br>SP_PIR_KEYWORDS	Base-excision repair, DNA replication.	3.39
GOTERM_BP_FAT	*in utero* embryonic development.	3.38
KEGG_PATHWAY<br>BIOCARTA<br>BBID	Cancer, Insulin signalling pathway, PTEN dependent cell cycle arrest and apoptosis.	3.35
GOTERM_BP_FAT<br>UP_SEQ_FEATURE<br>INTERPRO<br>GOTERM_MF_FAT	Negative regulation of gene expression, NAD binding.	3.29
GOTERM_BP_FAT	Induction of apoptosis by extracellular signals.	3.19
GOTERM_BP_FAT	DNA geometric change.	3.13
GOTERM_BP_FAT	Macromolecular complex assembly.	3.07
SP_PIR_KEYWORDS<br>GOTERM_MF_FAT<br>PIR_SUPERFAMILY<br>KEGG_PATHWAYS<br>INTERPRO<br>UP_SEQ_FEATURE<br>SMART<br>BIOCARTA	Serine/threonine-protein kinase, MAP kinase activity, SAP kinase activity, BCR signalling pathway.	3.03
GOTERM_BP_FAT	Regulation of homeostatic process, female sex differentiation, positive regulation of myeloid cell differentiation, T cell homeostasis.	2.97
GOTERM_BP_FAT	Negative regulation of protein modification process, regulation of membrane potential.	2.96
GOTERM_BP_FAT	Reproductive process in a multicellular organism, reproductive developmental process, sexual reproduction.	2.95

GOTERM_BP_FAT<br>KEGG_PATHWAY	Negative regulation of binding, regulation of transcription factor activity.	2.79
GOTERM_BP_FAT	Negative regulation of neuron apoptosis, cellular chemical homeostasis, regulation of membrane potential.	2.75
GOTERM_BP_FAT<br>GOTERM_MF_FAT<br>SP_PIR_KEYWORDS	DNA topoisomerase activity, DNA topological change.	2.75
KEGG_PATHWAY<br>BIOCARTA<br>GOTERM_BP_FAT	Signalling pathways (ErbB, TPO, IL 2, MAPKinase, Insulin, EGF, IL 3, BCR, IL 6), epidermal growth factor receptor signaling pathway.	2.75
UP_SEQ_FEATURE<br>INTERPRO<br>SMART	PIK-related kinase.	2.70
GOTERM_BP_FAT	Insulin receptor signalling pathway, regulation of glucose transport.	2.66
GOTERM_BP_FAT	Positive regulation of multicellular organism growth.	2.65
GOTERM_BP_FAT	Regulation of JNK cascade, regulation of stress-activated protein kinase signaling pathway.	2.62
GOTERM_BP_FAT<br>KEGG_PATHWAY<br>BIOCARTA	Insulin receptor signalling pathway, Trka receptor signalling pathway.	2.57
GOTERM_BP_FAT	Regulation of mitochondrial membrane potential, regulation of mitochondrial membrane permeability.	2.56
GOTERM_BP_FAT	Cell morphogenesis involved in neuron differentiation, neuron development.	2.53
GOTERM_BP_FAT	Blood vessel development.	2.52
GOTERM_BP_FAT	Positive regulation of immune system process, B cell differentiation, regulation of steroid metabolic process.	2.51

E.Score - Enrichment Score

Table S4 – List of candidate drugs for lifespan extension. Drugs colored green are experimentally validated

Drug	Main target function	Proposed/Used for	P-Value	Interactions with lifespan-extending genes	Total No: of Genes
Dacinostat	Histone Deacetylase inhibitor(Ganai, 2015)	Cancer	3.72826E-17	10	10
Givinostat	Histone Deacetylase inhibitor(Rambaldi et al., 2010)	Cancer	3.72826E-17	10	10
PCI-24781 (Abexinostat)	Histone Deacetylase inhibitor(Salvador et al., 2013)	Cancer	3.72826E-17	10	10
Belinostat	Histone Deacetylase inhibitor(Steele et al., 2008)	Cancer	4.00865E-16	10	11
Vorinostat	Histone Deacetylase inhibitor(Marks & Breslow, 2007)	Cancer	4.00865E-16	10	11
Pivanex	Histone Deacetylase inhibitor(Reid et al., 2004)	Cancer	7.29936E-14	8	8
Sodium phenylbutyrate	Histone Deacetylase inhibitor(Gilbert et al., 2001)	Cancer	7.29936E-14	8	8
Panobinostat	Histone Deacetylase inhibitor(Ellis et al., 2008)	Cancer	1.35939E-12	10	18
Valproic acid	Histone Deacetylase inhibitor & GABA activator(Phiel et al., 2001)	Cancer & Epilepsy	1.21378E-10	9	19
FK-228*	Histone Deacetylase inhibitor(Piekarz et al., 2009)	Cancer	6.24073E-09	5	5
CHR-3996	Histone Deacetylase inhibitor(Banerji et al., 2012)	Cancer	2.73968E-07	4	4
Choline	Neurotransmitter precursor	Vitamin	6.94313E-07	2	7
Entinostat	Histone Deacetylase inhibitor(Pili et al., 2012)	Cancer	1.33864E-06	4	5
GDC-0068	Serine/Threonine Kinase (Akt) inhibitor(Lin et al., 2013)	Cancer	1.20062E-05	3	3
MK-2206	Serine/Threonine Kinase (Akt) inhibitor(Sangai et al., 2012)	Cancer	1.20062E-05	3	3
Everolimus	mTOR inhibitor(Culp & Wood, 2009)	Cancer	1.74891E-05	4	8
Minaprine	Monoamine Oxidase inhibitor(Gijsman, Geddes, Rendell, Nolen, & Goodwin, 2004)	Depression	3.07631E-05	4	9
Tegaserod	5-Hydroxytryptamine receptor 4 (5-HT4) agonist, Serotonin antagonist(Müller-Lissner et al., 2001)	Irritable bowel syndrome	4.69291E-05	3	4
Romidepsin*	Histone Deacetylase inhibitor(Piekarz et al., 2009)	Cancer	7.69404E-05	4	11
Clodronate	ADP/ATP translocase inhibitor(Diel et al., 1998)	Osteoporosis & Cancer	0.000114645	3	5

*DGIdb erroneously considers FK-228 and Romidepsin as two distinct compounds. In green are drugs previously shown to extend lifespan: Sodium phenylbutyrate (Kang, Benzer, & Min, 2002), valproic acid (Evason, Collins, Huang, Hughes, & Kornfeld, 2008) and everolimus (Spindler, Li, Dhahbi, Yamakawa, & Sauer, 2012).

Table S5 - Categorization of DGIdb's drug-gene interactions into Anti/Pro/Neither classes

Anti	Pro	Neither (Not considered)
inhibitor	potentiator	n/a
antagonist	agonist	binder
blocker	inducer	antibody
inverse agonist	cofactor	other/unknown
antisense	product of	allosteric modulator
negative modulator	stimulator	multitarget
antisense oligonucleotide	activator	modulator
suppressor	partial agonist,agonist	agonist,antagonist
partial antagonist	chaperone	ligand
antagonist, inhibitory allosteric modulator	partial agonist	antagonist, partial agonist
inhibitor, antagonist	modulator,agonist	vaccine
antagonist, multitarget	positive allosteric modulator	adduct
cleavage	agonist, partial agonist	immunotherapy
multitarget, antagonist		antagonist, agonist
inhibitory allosteric modulator		partial agonist, antagonist
inhibitor, competitive		

Tables S6-S17 (in Excel files)

Table S6- Overlap analysis per class - Genome
Table S7- Overlap analysis per disease - Genome
Table S8- Overlap analysis per class - Genome with Publication Bias Correction
Table S9- Overlap analysis per disease - Genome with Publication Bias Correction
Table S10- Overlap analysis per class - Interactome
Table S11- Overlap analysis per disease - Interactome
Table S12- Overlap analysis per class - Interactome with Publication Bias Correction
Table S13- Overlap analysis per disease - Interactome with Publication Bias Correction
Table S14- Overlap analysis per class - including first order partners
Table S15- Overlap analysis per disease - including first order partners
Table S16- Overlap analysis per class - including co-expressed genes
Table S17- Overlap analysis per disease - including co-expressed genes

The tables present the aging gene sets in the columns and disease classes or diseases in rows. For each overlap between an aging gene set and a disease or a diseases class, there are two values, a p-value and the number of CAD-genes. These tables also present a colour code which corresponds to a green background behind the p-value if it is lower than 0.05 - meaning that is a significant value - and with a green background behind CAD-genes if their number is higher than the expected number by chance (not showed); for the both mentioned conditions, if the condition is not verified the background is orange. Only the overlaps with a higher number of CAD-genes than the CAD-genes expected by chance and a p-value lower than 0.05 are considered significant.

Table S18 - Number of genes of aging gene sets

Data Set	Initial Set	PBC 10+ Genome	Interactome	PBC 10+ Interactome	PBC 10+ With partners	PBC 10+ With RNAseq co-expressed genes
Human aging	298	253	294	251	7442	5109
All orthologues						
anti-longevity	448	216	408	214	3792	3942
pro-longevity	421	238	394	237	5628	4557
M. musculus						
anti-longevity	23	17	22	17	730	31
pro-longevity	59	49	57	48	2483	215
D. melanogaster						
anti-longevity	48	25	42	25	1131	77
pro-longevity	87	57	85	57	2855	577
C. elegans						
anti-longevity	381	185	344	183	2994	3545
pro-longevity	290	144	267	144	3286	2649
S. Cerevisiae						
anti-longevity	41	21	38	21	982	28
pro-longevity	13	12	13	12	298	194

Table S19 - Number of diseases at different thresholds for minimum number of genes

Threshold	Num. of diseases
10	130
20	65
30	44
40	25
50	21

Table S20 - Diseases list with the disease class and the number of genes. All shown diseases were analysed per class and diseases marked with asterisk (*) were also analysed individually.

Disease MSH Term	MeSH Disease Class	Num. of Genes
*Hypertension	Cardiovascular	172
*Myocardial Infarction	Cardiovascular	132
*Coronary Disease	Cardiovascular	104
Coronary Artery Disease	Cardiovascular	90
Hypertrophy, Left Ventricular	Cardiovascular	20
Thrombosis	Cardiovascular	22
*Arteriosclerosis	Cardiovascular	45
Diabetic Angiopathies	Cardiovascular	38
*Atherosclerosis	Cardiovascular	31
Graves Disease	Eye	32
Macular Degeneration	Eye	21
Diabetic Retinopathy	Eye	26
*Autoimmune Diseases	Immune System	20
*Hypersensitivity, Immediate	Immune System	32
*Arthritis, Rheumatoid	Musculoskeletal	80
*Osteoporosis	Musculoskeletal	30
*Lung Neoplasms	Neoplasms	92
*Stomach Neoplasms	Neoplasms	66
*Breast Neoplasms	Neoplasms	121
*Prostatic Neoplasms	Neoplasms	90
*Adenocarcinoma	Neoplasms	66
Skin Neoplasms	Neoplasms	28
*Coloretal Neoplasms	Neoplasms	96
Melanoma	Neoplasms	23
Esophageal Neoplasms	Neoplasms	38
Head and Neck Neoplasms	Neoplasms	39
Ovarian Neoplasms	Neoplasms	30
Urinary Blandder Neoplasms	Neoplasms	38
Liver Neoplasms	Neoplasms	30
Mouth Neoplasms	Neoplasms	31
Uterine Cervical Neoplasms	Neoplasms	21
Endometrial Neoplasms	Neoplasms	24
*Alzheimer Disease	Nervous System	115
*Parkinson Disease	Nervous System	50
*Multiple Sclerosis	Nervous System	49
*Diabetes Mellitus, Type 1	Nutritional and Metabolic	100
*Diabetes Mellitus, Type 2	Nutritional and Metabolic	219
*Obesity	Nutritional and Metabolic	127
*Asthma	Respiratory Tract	122
Pulmonary Disease, Chronic Obstruction	Respiratory Tract	33

Supplementary Figures

Figure S1: Similar top functional annotation clusters from the LongevityMap in two backgrounds. (Higher ranked clusters obtained from default background also have higher rank when using LongevityMap genes as background)

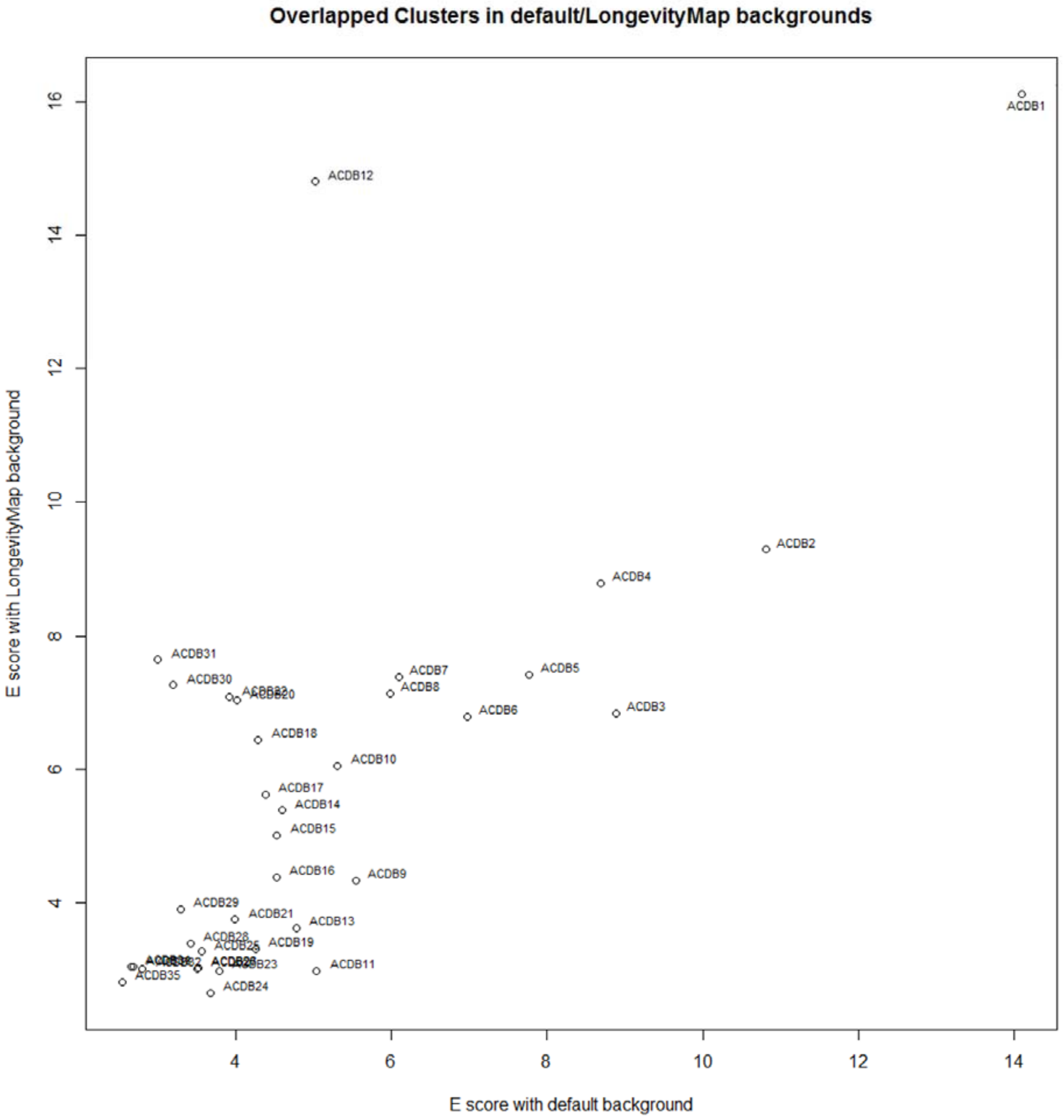

Figure S2: Analysis of publications number per gene for the human genome. This graph shows the variation of the number of genes for different thresholds of publications per gene. Thresholds between 8 and 20 were assessed.

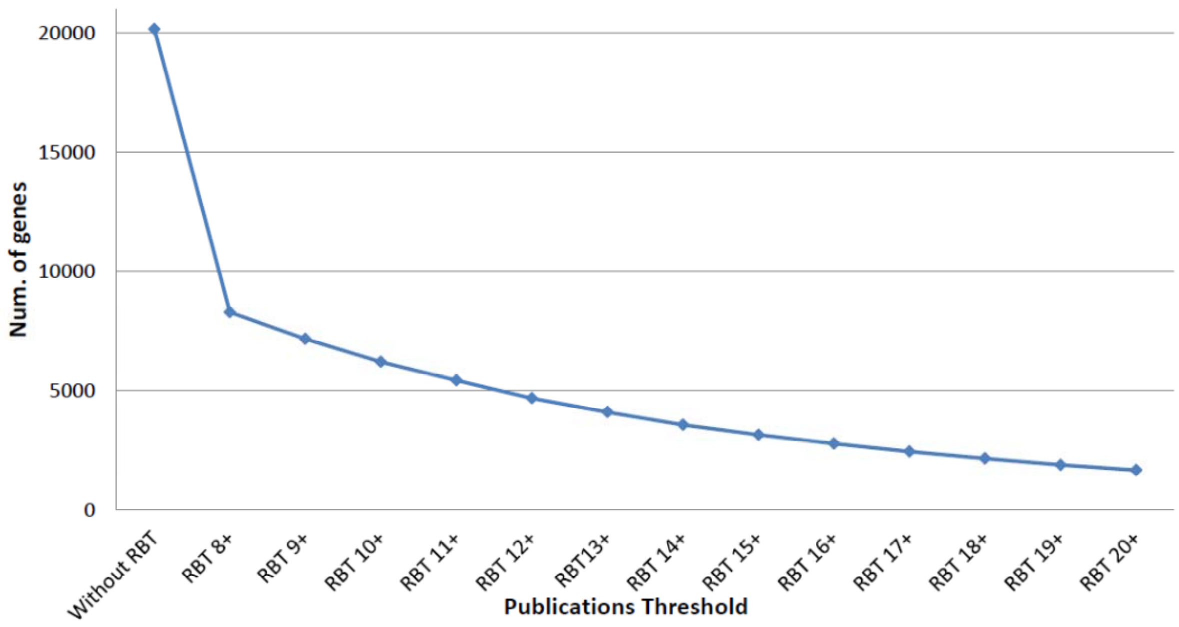

Figure S3: Number of significant diseases per gene set - Interactome without and with PBC. The graph on top summarizes the number of significant overlapping diseases per gene set in analysis without PBC and the graph on the bottom is relative to the analysis with PBC, both using the interactome as background without and with PBC, respectively. Different colours in each column correspond to each class of diseases. Above each column is the number of genes in the aging gene set.

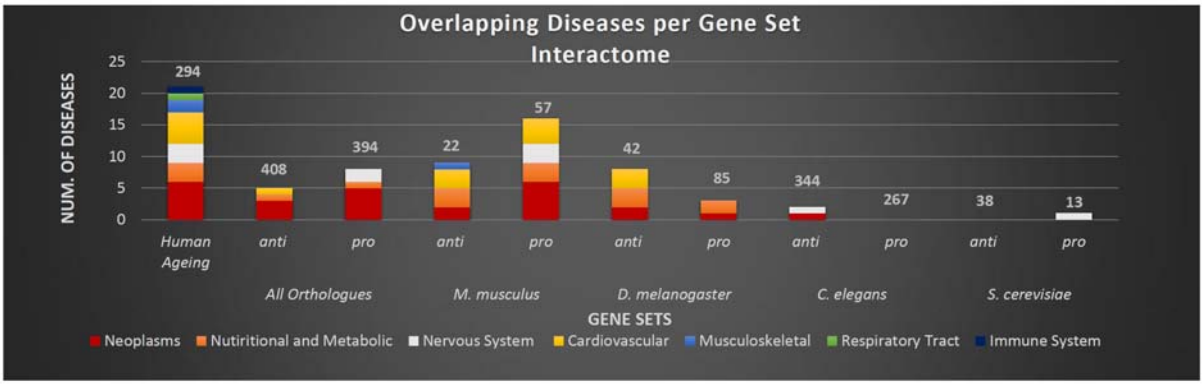

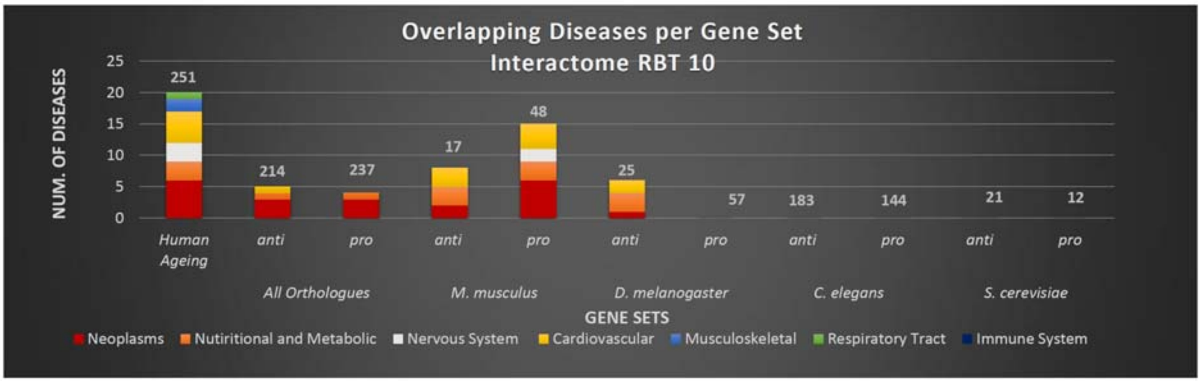

Supplementary References:

Banerji, U., Van Doorn, L., Papadatos-Pastos, D., Kristeleit, R., Debnam, P., Tall, M., … Eskens, F. a L. M. (2012). A phase I pharmacokinetic and pharmacodynamic study of CHR-3996, an oral class I selective histone deacetylase inhibitor in refractory solid tumors. *Clinical Cancer Research*, *18*(7), 2687–2694. http://doi.org/10.1158/1078-0432.CCR-11-3165

Culp, S. H., & Wood, C. G. (2009). Re: Efficacy of Everolimus in Advanced Renal Cell Carcinoma: A Double-Blind, Randomized, Placebo-Controlled Phase III Trial. *European Urology*, *55*(6), 1484–1485. http://doi.org/10.1016/j.eururo.2009.03.035

Diel, I. J., Solomayer, E. F., Costa, S. D., Gollan, C., Goerner, R., Wallwiener, D., … Bastert, G. (1998). Reduction in new metastases in breast cancer with adjuvant clodronate treatment. *The New England Journal of Medicine*, *339*(6), 357–63. http://doi.org/10.1056/NEJM199808063390601

Ellis, L., Pan, Y., Smyth, G. K., George, D. J., McCormack, C., Williams-Truax, R., … Prince, H. M. (2008). Histone Deacetylase Inhibitor Panobinostat Induces Clinical Responses with Associated Alterations in Gene Expression Profiles in Cutaneous T-Cell Lymphoma. *Clinical Cancer Research*, *14*(14), 4500–4510. http://doi.org/10.1158/1078-0432.CCR-07-4262

Evason, K., Collins, J. J., Huang, C., Hughes, S., & Kornfeld, K. (2008). Valproic acid extends Caenorhabditis elegans lifespan. *Aging Cell*, *7*(3), 305–317. http://doi.org/10.1111/j.1474-9726.2008.00375.x

Ganai, S. A. (2015). Strategy for enhancing the therapeutic efficacy of histone deacetylase inhibitor dacinostat: the novel paradigm to tackle monotonous cancer chemoresistance. *Archives of Pharmacal Research*. http://doi.org/10.1007/s12272-015-0673-9

Gijsman, H. J., Geddes, J. R., Rendell, J. M., Nolen, W. A., & Goodwin, G. M. (2004). Antidepressants for Bipolar Depression: A Systematic Review of Randomized, Controlled Trials. *American Journal of Psychiatry*, *161*(9), 1537–1547. http://doi.org/10.1176/appi.ajp.161.9.1537

Gilbert, J., Baker, S. D., Bowling, M. K., Grochow, L., Figg, W. D., Zabelina, Y., … Carducci, M. A. (2001). A Phase I Dose Escalation and Bioavailability Study of Oral Sodium Phenylbutyrate in Patients with Refractory Solid Tumor Malignancies A Phase I Dose Escalation and Bioavailability Study of Oral Sodium Phenylbutyrate in Patients with Refractory Solid, *7*(August), 2292–2300.

Kang, H.-L., Benzer, S., & Min, K.-T. (2002). Life extension in Drosophila by feeding a drug. *Proceedings of the National Academy of Sciences of the United States of America*, *99*, 838–843. http://doi.org/10.1073/pnas.022631999

Lin, J., Sampath, D., Nannini, M. a., Lee, B. B., Degtyarev, M., Oeh, J., … Lin, K. (2013). Targeting activated Akt with GDC-0068, a novel selective Akt inhibitor that is efficacious in multiple tumor models. *Clinical Cancer Research*, *19*(7), 1760–1772. http://doi.org/10.1158/1078-0432.CCR-12-3072

Marks, P. a, & Breslow, R. (2007). Dimethyl sulfoxide to vorinostat: development of this histone deacetylase inhibitor as an anticancer drug. *Nature Biotechnology*, *25*(1), 84–90. http://doi.org/10.1038/nbt1272

Müller-Lissner, S. a, Fumagalli, I., Bardhan, K. D., Pace, F., Pecher, E., Nault, B., & Rüegg, P. (2001). Tegaserod, a 5-HT(4) receptor partial agonist, relieves symptoms in irritable bowel syndrome

patients with abdominal pain, bloating and constipation. *Alimentary Pharmacology & Therapeutics*, *15*(10), 1655–1666. http://doi.org/apt1094 [pii]

Phiel, C. J., Zhang, F., Huang, E. Y., Guenther, M. G., Lazar, M. A., & Klein, P. S. (2001). Histone Deacetylase Is a Direct Target of Valproic Acid, a Potent Anticonvulsant, Mood Stabilizer, and Teratogen. *Journal of Biological Chemistry*, *276*(39), 36734–36741. http://doi.org/10.1074/jbc.M101287200

Piekarz, R. L., Frye, R., Turner, M., Wright, J. J., Allen, S. L., Kirschbaum, M. H., … Bates, S. E. (2009). Phase II Multi-Institutional Trial of the Histone Deacetylase Inhibitor Romidepsin As Monotherapy for Patients With Cutaneous T-Cell Lymphoma. *Journal of Clinical Oncology*, *27*(32), 5410–5417. http://doi.org/10.1200/JCO.2008.21.6150

Pili, R., Salumbides, B., Zhao, M., Altiok, S., Qian, D., Zwiebel, J., … Rudek, M. a. (2012). Phase I study of the histone deacetylase inhibitor entinostat in combination with 13-cis retinoic acid in patients with solid tumours. *British Journal of Cancer*, *106*(1), 77–84. http://doi.org/10.1038/bjc.2011.527

Rambaldi, A., Dellacasa, C. M., Finazzi, G., Carobbio, A., Ferrari, M. L., Guglielmelli, P., … Barbui, T. (2010). A pilot study of the Histone-Deacetylase inhibitor Givinostat in patients with JAK2V617F positive chronic myeloproliferative neoplasms. *British Journal of Haematology*, no–no. http://doi.org/10.1111/j.1365-2141.2010.08266.x

Reid, T., Valone, F., Lipera, W., Irwin, D., Paroly, W., Natale, R., … Bhatnagar, A. (2004). Phase II trial of the histone deacetylase inhibitor pivaloyloxymethyl butyrate (Pivanex, AN-9) in advanced non-small cell lung cancer. *Lung Cancer*, *45*, 381–386. http://doi.org/10.1016/j.lungcan.2004.03.002

Salvador, M. a., Wicinski, J., Cabaud, O., Toiron, Y., Finetti, P., Josselin, E., … Ginestier, C. (2013). The histone deacetylase inhibitor abexinostat induces Cancer stem cells differentiation in breast Cancer with low Xist expression. *Clinical Cancer Research*, *19*(23), 6520–6531. http://doi.org/10.1158/1078-0432.CCR-13-0877

Sangai, T., Akcakanat, A., Chen, H., Tarco, E., Wu, Y., Do, K. A., … Meric-Bernstam, F. (2012). Biomarkers of rsponse to Akt inhibitor MK-2206 in breast cancer. *Clinical Cancer Research*, *18*(20), 5816–5828. http://doi.org/10.1158/1078-0432.CCR-12-1141

Sleigh, S. H., & Barton, C. L. (2010). Repurposing Strategies for Therapeutics. *Pharmaceutical Medicine*, *24*(3), 151–159. http://doi.org/10.1007/BF03256811

Spindler, S. R., Li, R., Dhahbi, J. M., Yamakawa, A., & Sauer, F. (2012). Novel protein kinase signaling systems regulating lifespan identified by small molecule library screening using Drosophila. *PloS One*, *7*(2), e29782. http://doi.org/10.1371/journal.pone.0029782

Steele, N. L., Plumb, J. a, Vidal, L., Tjørnelund, J., Knoblauch, P., Rasmussen, A., … DeBono, J. S. (2008). A phase 1 pharmacokinetic and pharmacodynamic study of the histone deacetylase inhibitor belinostat in patients with advanced solid tumors. *Clinical Cancer Research : An Official Journal of the American Association for Cancer Research*, *14*(6), 804–810. http://doi.org/10.1158/1078-0432.CCR-07-1786
